# Latent variable modeling to develop a robust proxy for sensitive behaviors: application to latrine use behavior and its association with sanitation access in a middle-income country

**DOI:** 10.1186/s12889-018-6373-x

**Published:** 2019-01-19

**Authors:** V. K. Lopez, B. T. West, P. J. Clarke, E. Quentin, J. N. S. Eisenberg

**Affiliations:** 1 Department of Epidemiology, University of Michigan School of Public Health, 1415 Washington Heights, Ann Arbor, MI 48109 USA; 2 Institute for Social Research, University of Michigan, Ann Arbor, MI USA; 3 Instituto Nacional de Investigación en Salud Pública, Centro de Investigación EpiSIG, Quito, Ecuador

**Keywords:** Sensitive behaviors, Sanitation, Latrine use, Latent class analysis, Measurement

## Abstract

**Background:**

Globally, diarrhea is a leading cause of child morbidity and mortality. Although latrines are integral for reducing enteric pathogen transmission, several studies have shown no evidence that latrine ownership improved child health. There are a number of explanations for these results. One explanation is that latrine access does not equate to latrine use. Latrine use, however, is difficult to accurately ascertain, as defecation behavior is often stigmatized. To address this measurement issue, we measure latrine use as a latent variable, indicated by a suite of psychosocial variables.

**Methods:**

We administered a survey of 16 defecation-related psychosocial questions to 251 individuals living in rural Ecuador. We applied latent class analysis (LCA) to these data to model the probability of latrine use as a latent variable. To account for uncertainty in predicted latent class membership, we used a pseudo-class approach to impute five different probabilities of latrine use for each respondent. Via regression modeling, we tested the association between household sanitation and each imputed latrine use variable.

**Results:**

The optimal model presented strong evidence of two latent classes (entropy = 0.86): consistent users (78%) and inconsistent users (22%), predicted by 5 of our 16 psychosocial variables. There was no evidence of an association between the probability of latrine use, predicted from the LCA, and household access to basic sanitation (OR = 1.1, 95% CI = 0.6–2.1). This suggests that home access to a sanitation facility may not ensure the use of the facility for every family member at all times.

**Conclusion:**

Effective implementation and evaluation of sanitation programs requires accurate measurement of latrine use. Psychosocial variables, such as norms, perceptions, and attitudes may provide robust proxy-measures. Future longitudinal studies will help to strengthen the use of these surrogate measures, as many of these factors may be subject to secular trends. Additionally, subgroup analyses will elucidate how our  proxy indicators of latrine defecation vary by individual-level characteristics.

**Electronic supplementary material:**

The online version of this article (10.1186/s12889-018-6373-x) contains supplementary material, which is available to authorized users.

## Background

Diarrheal disease, which is both preventable and treatable, is a persistent leading cause of morbidity and mortality for children under 5 years of age throughout the globe. Diarrhea-related mortality accounts for nearly 500,000 child deaths per year, which are concentrated in resource-limited settings [1]. Millions of children worldwide are undernourished [2], and while malnutrition has a host of causes, enteric pathogen exposure is associated with both acute and chronic malnutrition [3, 4]. Water, sanitation, and hygiene (WASH) interventions reduce exposure to enteric pathogens that cause illness [5] and it is estimated that diarrheal-related child mortality could be reduced by more than one-third in low and middle-income countries (LMICs) if sanitation interventions were implemented at full-scale [6]. Sanitation blocks multiple transmission routes of enteric pathogens, reducing individual-level exposures as well as community-level exposures [7, 8]. Indeed, sanitation programs are viewed by governments as fundamental for achieving Sustainable Development Goal (SDG) 6.2, that is, to end open defecation and provide universal access to adequate and equitable sanitation [9]. Yet, results from two recent studies of latrine construction interventions did not find an effect on child health; one rationale for these results is limited uptake of latrine use [10, 11]. Moreover, an analysis of Demographic and Health Survey data has shown a null association between access to improved sanitation facilities and prevalence of diarrhea in children throughout much of the globe [12]. These results suggest a need to emphasize latrine use in programmatic sanitation interventions. Like others, we suggest that WASH program success can be better evaluated if behavior is incorporated as an indicator of WASH success rather than simply the presence of sanitation hardware [13].

Historically, sanitation intervention success has been measured as a the presence of a household sanitation facility (traditionally and synonymously referred to as “sanitation access” within the development field and the focus of the SDGs) rather than individual sanitation practices [14]. One reason that toilet presence has been the accepted exposure variable is that it is relatively easy to measure. Latrine use, however, is difficult to measure and no gold standard of measurement exists. A limited number of intervention studies include a metric of defecation behavior, and the methods used to capture latrine use behavior vary across studies [15]. The most commonly used measure is self-reported defecation practices; however, outside social contexts where report of open defecation is not stigmatized, such as in India [16], survey questions asking about a respondent’s sanitation behaviors present challenges, such as social desirability bias due to the sensitivity of the topic [17, 18]. More recent literature has sought to move beyond a binary self-report of defecation practices, such as Jenkins et al.’s 2014 scale of defecation [19]; or Dreibelbis et al.’s 2015 [20] and Lopez et al.’s 2019 [21] use of psychosocial factors to predict latrine use. Dreibelbis et al.’s study and Lopez et al.’s study, in particular, are promising examples of integrating social theory and quantitative assessment of WASH behavior. These studies, however, rely on self-reported defecation behaviors and/or report of other’s defecation behaviors as outcomes. Such limitations potentially add misclassification and bias into their study results.

Beyond self-report or proxies of population-level psychosocial factors, hygiene behavior is commonly captured through direct observation [22, 23], which is a time-consuming effort subject to the Hawthorne effect [24]. Other measures of behavior include use of sensors inside a latrine to quantify the number of times a latrine was entered [25] and observations of the latrine to assess whether it appears to have been used [26]. While sensors may supply a more objective estimate of overall latrine use than self-report [27], neither sensors nor observations provide information necessary for assessing individual-level latrine use, or the proportion of users on the household or community levels. Such details are integral for understanding exposure risk of negative health outcomes, such as diarrheal disease and undernutrition.

This measurement conundrum for latrine use behavior is not unique to the sanitation field. Rather, many sub-fields of public health that rely on self-report of sensitive behaviors face similar issues of misclassification and measurement error. For example, epidemiology studies focusing on sexually transmitted infections have long been challenged by measurement of behavior [28], with self-reported condom use, a key intervention, presenting misclassification within the study [29, 30]. Here, we take a latent variable approach to capturing latrine use in a LMIC population with high latrine access, variable latrine use, and stigmatized self-report of defecation practices. Our approach is rooted in health behavior theory that recognizes both demographic factors and social processes as determinates of individual-level behavior [31]. Using psychosocial indicators of latrine use behavior, we first apply latent variable modeling to create a proxy-indicator of latrine use at the individual level. Latent class modeling specifically refers to a group of techniques that identify one’s underlying propensities to respond to particular questions and classifies a person into a group given their responses [32]; thus, it lends itself well to measuring sensitive concepts that may not be easily captured by one direct question. We hypothesize that individuals in this population will generally belong to one of three classes: those who always use a latrine for defecation, those who sometimes use a latrine for defecation, and those who never use a latrine. With an assigned likelihood of latrine use, we next test the association between the latent variable measure of latrine use and household-level access to an improved, non-shared sanitation facility.

## Methods

### Data

The data are from a cross-sectional sample as part of a longitudinal study examining the relationship between latrine use and child health. Data were collected between July and August 2016, which is the dry season of the geographical study site. We surveyed 251 individuals (in 98 households) 13 years and older living in 20 rural communities in Esmeraldas, Ecuador (see Table 1 for demographics). The communities selected for enrollment participated in previous enteric pathogen transmission studies [33]. Eligibility for household enrollment was based on whether a child 12 month of age or younger lived in the home. Within each selected community, a household census was conducted to determine if eligibility criteria was met; if so, the household was approached for participation. Of eligible households, 94% were contacted and of these, 94% participated. Each individual 13 years or older living in an enrolled household was also approached for participation. Ninety-two percent of eligible individuals were present during the field team’s visits and 81% of them consented to interview. The study communities are heterogeneous in terms of race/ethnicity; there are Afro-Ecuadorians, Chachi (an indigenous groups), and *mestizos*.

**Table 1:** Background characteristics of the study sample

Background characteristic	Mean (SD) / Proportion	N
Age (in years)	23 (14)	251
Sex		
Female	0.65	162
Male	0.35	89
Ethnicity		
Afro-Ecuadorian	0.63	157
Mestizo and Other	0.13	33
Chachi	0.24	61
Educational Attainment		
Less than primary school	0.25	63
Completed primary school	0.20	51
Less than secondary school	0.36	90
Completed secondary school	0.17	43
Missing	0.02	4
Payment Type Received for Employment		
Solely cash	0.47	118
Cash and kind/ solely kind	0.06	16
Not paid	0.16	41
Not employed in the previous 12 months	0.30	76
Living in Household with Cement Walls		
Yes	0.45	115
No	0.51	128
Missing	0.03	8
Living in an Asset-Deprived Household		
Yes	0.61	152
No	0.39	98
Missing	< 0.01	1
Household Sanitation Access		
Improved Access		
Basic sanitation	0.53	132
Shared sanitation	0.33	118
Unimproved Access	0.05	13
No access to a sanitation facility	0.09	22
Missing	0.01	2

Median (SD) is reported for age. For the other covariates, the frequency distribution (in proportions) is reported. The corresponding number of individuals (N) is also shown

### Latrine use for defecation

Individuals in our study site practice a variety of sanitation behaviors. They range from private household latrine use, to public community latrine use, to open defecation [21]. Defecation practices vary by demographic factors such as individual age, gender, place in the family hierarchy, and time spent outside of the home, as well as external factors, such as personal safety and privacy. External factors influencing defecation extend beyond the individual level. Given the tropical climate of the study site, rainfall also influences when and where one defecates. Details regarding multidimensional drivers of latrine use for defecation are presented elsewhere [21].

### Measures

Study participants were interviewed face-to-face by a local, trained survey team within the participant’s home. The interviews focused on the psychosocial determinants of defecation within latrines and sociodemographic information. The survey tool was designed using the Integrated Behavior Model of Water Sanitation and Hygiene (IBM-WASH), a health behavior framework that presents behavior as a byproduct of intersecting dimensions of technology, context, and psychosocial factors that operate through ecological levels [34]. We applied the IBM-WASH framework to develop indicators reflecting determinants of latrine defecation behavior as we conceptualize human behavior to be dynamic influenced by a multidimensional ecosystem.

The survey tools consisted of 16 indicators, each measured by a specific question, to assess psychosocial determinants of latrine use for defecation (Table 2). Each survey question had three possible responses: *yes*, *no*, or *don’t know*. We determined these indicators using ethnographic data from the study site as well as their demonstrated association with consistent defecation in a latrine in the communities of study. Details are summarized in a prior publication [21]. Some of these indicators reflect descriptive social norms of defecation behavior in the communities; for example, whether a respondent agrees or disagrees that men in their village use a latrine during the dry season. Other indicators reflect themes of personal safety, convenience, and anxiety related to latrine use, as well as attitudes towards sharing a latrine with others. We also asked about perceptions of the latrine cleanliness and maintenance, and about habitual latrine use.

**Table 2:** Descriptive statistics for latent class analysis indicators: the proportion of respondents that agree, disagree, or answer don’t know to each survey statement

LCA Indicators			
Survey Questions	Proportion of Agreement	Proportion of Disagreement	Proportion of Don’t Know
When I use the latrine, it causes me to feel anxious.	0.19	0.82	0.00
I use the latrine every day.	0.91	0.09	0.00
I do not use the latrine when it is raining because I do not want to get wet.	0.81	0.19	0.00
During the dry season, I think that most of the men in my village regularly use a latrine.	0.68	0.14	0.18
During the rainy season, I think all of my neighbors regularly use a latrine.	0.80	0.07	0.14
During the rainy season, I think that most of the children in my village regularly use a latrine.	0.87	0.08	0.04
There are too many people in this household for one latrine.	0.43	0.56	0.01
If my household did not have its own latrine, I would use my neighbor’s latrine.	0.86	0.14	0.00
The cabin of the latrine is too small for me to use.	0.26	0.73	0.01
I am pleased with how the latrine looks.	0.64	0.35	0.01
The latrine’s basin is strong enough to hold my weight.	0.92	0.06	0.01
The latrine is clean enough to use.	0.87	0.10	0.03
It is more convenient to defecate outside than to return home to use the latrine.	0.44	0.55	0.00
My morning routine is not suited for using the latrine to defecate.	0.33	0.66	0.01
It is more convenient to use the latrine at night than to defecate in a container within my household.	0.85	0.15	0.01
It is dangerous to use the latrine at night.	0.36	0.64	0.00

The survey team documented the presence of a sanitation facility following WHO/UNICEF Joint-Monitoring Programme definitions. A sanitation facility is considered improved if it separates human excreta from contact with the facility user [35]. Improved sanitation facilities that are not shared with other households are defined as basic sanitation, whereas an improved facility that is shared with at least two households is considered limited sanitation. An unimproved facility does not provide hygienic separation of fecal matter from latrine users.

There are a number of important covariates for the analysis. Education and wealth, metrics of socioeconomic position [36], are associated with latrine access and the desire to own a latrine [37, 38]. Defecation practices, as well as the sanitation facility used, vary by race/ethnicity within the study site. Women and men display distinct patterns of latrine defecation that may not be captured by the other covariates [39, 40]. Because wealth ascertainment in LMIC research often reflects asset ownership, which proxies one’s access to resources [41], we measured asset ownership and whether one’s household walls were constructed with cement. Asset deprivation is a composite indicator considered within the Standard of Living component in the Multidimensional Poverty Index that accounts for access to three different types of assets: information, mobility, and livelihood [42]. Sociodemographic and asset covariates were measured via self-report; trained field staff observed household construction materials.

The Institutional Review Board at the University of Michigan and the Universidad San Francisco de Quito Bioethics Committee approved the data collection methods and study procedures.

### Analytical approach

We used latent class analysis (LCA) to classify individuals according to their propensity to use a latrine for defecation. LCA is a modeling approach that enables estimation of the probability of an individual belonging to one of a small number of hypothesized latent (or unobserved) classes based on a set of measured indicators, and allows for measurement error to be explicitly accounted for when estimating the final categorical latent construct [43]. To model latrine use as a latent construct, we used the 16 psychosocial determinants of latrine defecation measured in the aforementioned survey tool, and employed full information maximum likelihood (FIML) with robust standard errors (see Additional file 1 for polychoric correlations between indicators). Of note, because there is no gold standard metric for latrine defecation, we chose not to compare the latent assignment of latrine use for defecation to any other metric. Thus, this approach does not validate the final classifications. Rather, we assess for validity by examining the final model entropy, considering the item probability for each indicator, comparing these patterns to prior research conducted in the study site, and finally, by evaluating the final model with the Hill criteria for causality.

Model building proceeded in a sequential process by first specifying the measurement model, where the 16 measured determinants were indicators of latent class membership. We then specified the hypothesized number of latent classes, first including three classes and then including two classes. In the three-class model, we expect individuals to be grouped into the following latrine user categories: never users, occasional users, and always users. A two-class model aggregates these categories into latrine users and non-users or consistent latrine users and inconsistent latrine users. Interpretation of classes beyond three groups is challenging; it is difficult to disaggregate meaning results between theoretical “less than always latrine users” and “more than never latrine users”. To determine the optimal class solution (that is, the number of classes within the sample), we compared the model fit between the three-class and two-class models. Good fitting models are characterized by: [1] a low Bayesian Information Criterion (BIC) value; [2] a statistically non-significant chi-square goodness-of-fit statistic that assessed the observed class distribution and expected probability distribution of classes; and [3] relative entropy above 0.80 [44–47] (see Additional file 2 for a comparison of class assignment in each of the models tested). Once the optimal class solution was determined, we examined the latent-class conditional item probabilities for each indicator (i.e., the probability of response to each survey question given class assignment). Uninformative indicators were removed from the model (see Additional file 3 for conditional item probabilities for all indicators). An indicator was considered uninformative if the item probability confidence interval overlapped between class assignments. Please also see the Additional file 4 for gender-stratified LCA models.

In addition to identifying latent classes of latrine users, we also described the characteristics of the individuals in each class. Because of the inherent uncertainty in predicted class membership via LCA, we employed a multiple imputation (or pseudo-class) approach [48], using five random draws from a uniform distribution to generate five simulated class memberships for each respondent (given their original predicted class probability). Demographic descriptive statistics are presented for the modal class membership for each individual from the five imputations.

Using each imputed class membership as the outcome to test the association between class membership (the dependent variable) and household-level access to a basic sanitation facility, we fit five logistic regression models. Each of the regression models was adjusted for: individual-level educational attainment (categorical), race/ethnicity (binary, as Afro-Ecuadorian and non-Afro-Ecuadorian), and gender (binary). While it is theoretically plausible that gender acts as an effect modifier, it was not possible to explore this given the small sample size. At the household-level, the following variables were also included as proxy-indicators to control for wealth: cement household walls (binary) and whether a household is asset- deprived (binary). We accounted for household clustering when computing standard errors and planned to run multinomial logistic models if the three-class outcome was selected and binary logistic regression if the two-class outcome was chosen.

Following the regression modeling, estimates from the five models fitted to the imputed data sets were combined following rules described by Rubin [49] (see Additional file 5 for demographic proportions for each imputation and for model results for each imputation). Combining the models allows for the standard error of the association between latrine access and latrine use for defecation to be calculated.

Analyses were conducted using the R (version 3.4.1) packages *polycorr*, *poLCA*, and *survey*; see Additional file 6 for R code.

## Results

The study sample was young (median age = 23), predominately female (65%) and Afro-Ecuadorian (63%). Most participants did not complete high school (81%), and of those that worked in the last year, it was most common to receive cash remittance. Slightly more than half of respondents live in households with non-cement walls (51%) and in households with access to basic sanitation (53%). Sixty-one percent of participants were from asset-deprived homes, and overall access to improved sanitation is high in the population (86%), with half of the sample having access to a privately owned, improved sanitation facility (i.e., basic sanitation) (Table 1).

The responses to the 16 indicators are presented in Table 2. Survey questions asking about within-home latrine sharing and about convenience of latrine use while outside of the home had more evenly split responses relative to the rest of the survey questions. While most survey questions generated a *yes* or *no* response, indicators asking about descriptive norms of latrine use by men and neighbors tended to have more *don’t know* responses.

### Model fit

Using all 16 indicators, the 3-class and 2-class model solutions presented similar model fit statistics and predicted class memberships (Table 3). Nested group profiles are evident between the models. We thus label the three-class groups as “never users” (1%), “sometimes users” (25%), and “always users” (74%) and membership in the two-class model as “inconsistent latrine users” (22%) and “consistent latrine users” (78%). Within the 3-class model it is not feasible statistically to distinguish the 1% of the sample (*n* = 3, i.e., the “never users”) from the remainder in further multivariable analyses; thus, the 3-class solution was rejected.

**Table 3:** Model fit information and distribution of predicted classes probabilities using all 16 indicators

Number of classes	BIC	X2	Relative entropy	Predicted class probabilities
2	4675	2.9E+ 13	0.86	0.22; 0.78
3	4675	2.3E+ 06	0.85	0.01; 0.25; 0.74

After removing uninformative indicators from the two-class model, five indicators remained. The chi-squared (395) and BIC (1527) estimates of the 5-indicator 2-class model were drastically improved relative to the initial 16-indicator model (chi-squared = 2.9E+ 13 and BIC = 4675), while the entropy estimate (0.86) and predicted class memberships remained the same (0.78 and 0.22). The two-class model with five indicators was used in further analyses.

Overall, the final two-class model presents evidence of high conditional probabilities for four of the five indicators in the consistent latrine use class, indicating robust classification (Table 4). This is also reflected in the high value for the entropy estimate. The indicator for within-household latrine sharing (“*There are too many people in this household for just one latrine”*), however, has a low conditional item probability among those with assigned consistent latrine use membership. Only 52% of consistent latrine users responded *no* to this question, revealing that this indicator may not appropriately distinguish between classes.

**Table 4:** The parameter estimates for the final 2-class model that included 5 indicators: item conditional probability, standard error (SE), and response to the survey question

Indicator	Consistent Latrine Use		Inconsistent Latrine Use	
	Question Response	Item Probability (SE)	Question Response	Item Probability (SE)
During the dry season, I think that most of the men in my village regularly use a latrine.	Yes	0.85 (0.03)	Don’t Know	0.53 (0.11)
During the rainy season, I think all of my neighbors regularly use a latrine.	Yes	1.00 (0.00)	Don’t Know	0.57 (0.11)
During the rainy season, I think that most of the children in my village regularly use a latrine.	Yes	0.96 (0.02)	Yes	0.60 (0.08)
There are too many people in this household for one latrine.	No	0.52 (0.03)	No	0.70 (0.07)
If my household did not have its own latrine, I would use my neighbor’s latrine.	Yes	0.90 (0.02)	Yes	0.71 (0.07)

The overall probability of assignment into the consistent latrine use membership class is 0.78 while assignment into the inconsistent latrine use class was 0.22. The model fit statistics include BIC = 1527; X2 = 395; and relative entropy = 0.86

### Latent class membership assignment

Based on the final 2-class model, consistent latrine use was lowest among young adults (age 18–21), and also increased with higher educational attainment (Table 5). Consistent latrine use was also lowest among those that were unemployed in the prior year, while access to resources at the household-level showed no differences in latrine use. Accounting for individual factors and household resources, the fully adjusted regression model results showed no evidence of an association between household access to a basic sanitation facility and the probability of latrine use (Table 6; Basic Sanitation OR = 1.1, 95% CI = 0.6–2.1).

**Table 5:** The proportion of the population that are classified as consistent latrine users, by background characteristics

Demographic Variable	Proportion in Consistent Latrine Use Class (SE)	N
Age		
Age 13–17	0.80 (0.05)	58
Age 18–21	0.64 (0.08)	48
Age 22–26	0.80 (0.06)	49
Age 27–36	0.80 (0.06)	46
Age 37–83	0.81 (0.06)	50
Sex		
Females	0.75 (0.04)	162
Males	0.81 (0.04)	89
Ethnicity		
Afro-Ecuadorian	0.76 (0.04)	157
Mestizo and Other	0.78 (0.07)	33
Chachi	0.79 (0.06)	61
Educational Attainment		
Less than primary school	0.71 (0.06)	63
Completed primary school	0.75 (0.06)	51
Less than secondary school	0.80 (0.07)	90
Completed secondary school	0.81 (0.10)	43
Payment Type Received for Employment		
Solely cash	0.81 (0.04)	118
Cash and kind/ solely kind	0.88 (0.10)	16
Not paid	0.76 (0.07)	41
Not employed in the previous 12 months	0.70 (0.05)	76
Living in Household with Cement Walls		
Yes	0.74 (0.04)	115
No	0.78 (0.05)	128
Living in an Asset-Deprived Household		
Yes	0.74 (0.04)	152
No	0.80 (0.06)	98

Standard error (SE) and the total sample size for each demographic group are also presented

**Table 6:** Overall association between access to basic sanitation and latrine use (combined from each model using imputed latrine use as the outcome)

Model Covariate	Unadjusted OR (95% CI)	Adjusted OR (95% CI)
Less than basic sanitation *(referent group)*	1 (−)	1 (−)
Basic sanitation	1.1 (0.6–2.2)	1.1 (0.6–2.1)
Less than primary school *(referent group)*	–	1 (−)
Completed primary school	–	1.4 (0.6–3.3)
Less than secondary school	–	2.1 (0.9–4.9)
Completed secondary school	–	2.4 (0.8–6.8)
Chachi, Mestizo, or Other Ethnicity (*referent group*)	–	1 (−)
Afro-Ecuadorian	–	1.0 (0.5–2.0)
Male *(referent group)*	–	1 (−)
Female	–	1.5 (0.8–3.2)
Household walls constructed of other material (*referent group*)	–	1 (−)
Household constructed of cement	–	0.6 (0.3–1.2)
Asset Deprived Household (*referent group*)	–	1 (−)
Non-asset Deprived Household	–	0.5 (0.3–1.0)

The odds ratios (OR) and 95% confidence intervals (CI) is presented for relevant variables in the unadjusted and the adjusted models

## Discussion

For latrines to reduce pathogen transmission they must remain clean and be used. Thus, research and programmatic evaluation need to have accurate defecation behavior measurements. To this end, our findings reveal three important insights into the complicated relationship between latrine presence and latrine use. First, our model predicts that less than a quarter of this population practices inconsistent latrine defecation (22%, Table 2), even when access to improved sanitation is high (86%, Table 1). Although we do not present self-reported latrine use data, we suspect that misclassification would have been high in this variable given that less than 10% of respondents disagreed with the statement “I use a latrine daily” (see Additional file 7 for further comparisons). Additionally, we can also find insight from prior qualitative research on this topic. From qualitative work, we know that defecation behaviors besides latrine use are not uncommon and that self-reported defecation behaviors in a quantitative survey context are likely misclassified. The triangulation of misclassification in self-report latrine use for defecation and prior research in the study site suggests that psychosocial indicator variables are a potential surrogate for latrine defecation that minimizes misclassification bias. Second, we found no evidence that the presence of basic sanitation within the home was associated with latrine use. This result suggests that private, within-home sanitation does not ensure the use of said facility for every family member each time one needs to defecate. Our model result is consistent with data from India, which present variable sanitation practices among individuals living in a household with a sanitation facility [16, 50]. Some individuals will defecate in the household’s latrine, while others practice open defecation. Our null finding also provides an explanation for the null relationship between latrine access and child health observed in a number of settings [10–12]. And third, we found that five simple indicators could be used as reasonable indicators of the two latent classes of individuals in this population, as evidenced by a high entropy value for the final model that approached one (0.86). This high entropy value indicates limited misclassification in group assignment and is also an overall reflection of the indicators’ high conditional item probabilities in the consistent latrine use class [51, 52]. The strength of these indicators suggests that they are plausible underlying manifestations of latrine defecation. In further examining which indicators were informative for group classification, social theories of behavior provide additional rationale for these model results.

Of the 16 indicators examined, those that distinguished between class memberships asked individuals about community defecation norms (i.e., other’s latrine use for defecation) and latrine sharing between households. We also conducted a sensitivity analysis with these five societal-level indicators to determine whether they were useful in distinguishing between three classes of latrine users; the three-class model results were reasonable, however, the sample size of the groups was too small to permit well-powered comparisons. The strength of community level norm indicators to classify latent class membership, coupled with social theories of behavior, suggests potential of these indicators to serve as a proxy measure for individual behavior. Social theories of behavior view individual factors alone as limited metrics of behavior [53]. Sociocultural factors shaping daily habits in invisible ways [54]; humans often exhibit the behavior of those around them, where normative expectations playing a key role in one’s own actions [55]. For example, the LCA suggests that agreement with indicators asking whether others use latrines presents a very high probability that one is a consistent latrine user. Moreover, agreement with statements regarding latrine sharing between households may reflect a strong commitment to latrine use; that is, an individual implies that they would willingly face a variety of barriers to use someone else’s latrine (inconvenience, distance, dirty environment, etc.) if within-household access to a latrine did not exist. The psychological literature supports commitment as one important driver to behavior [56], again providing evidence of a plausible mechanism driving latrine defecation. If we apply traditional metrics of Hill’s exposure-outcome causality criteria to the study findings, our results exhibit strength, consistency (with the body of research), plausibility, and coherence [57]. Use of the Hill criteria provides evidence that these indicators are valid proxies of latrine defecation behavior.

Although the indicators used in the model were plausible, consistent, and coherent, our approach does not validate that the selected psychosocial variables are robust indicators of latrine use. Given the challenges of collecting unbiased measurements of latrine use, validating the predictive power of these indicators is difficult. Nevertheless, to address this limitation, we conducted extensive fieldwork to assess drivers of latrine defecation [21]. Beyond this initial work to define the 16 indicator variables that have potential to be surrogate indicators of latrine use, our LCA analysis suggests that community-level norms of latrine defecation are the strongest indicators. Thus, there is good potential for these indicators to generalize to other populations. Indeed the social environment tends to influence health behaviors [58]. Related to defecation, social norms and the behaviors of others in our social networks have been identified as determinants of latrine use behavior in India and Ethiopia [59–61]. The Community-Led Total Sanitation Campaign, a sanitation program targeting LMICs communities that seeks to end open defecation and has received lots of attention throughout the globe, operates through levels of behavior change that include community-pressure to promote latrine use [62, 63]. Our research was conducted in a very specific population; and it plausible that the latrine use and its drivers differ across the 20 communities included in our study as well as from drivers in other cultural contexts. Prior comparisons of the determinants of latrine defecation across cultures and geographical location highlight the role of shared community values, as well as perceptions of latrine cleanliness, on influencing behavior [21]. However, because prior studies are not void of self-report defecation behavior, it is plausible that identified determinants may be biased. Thus, determining which social norms of latrine defecation are applicable indicators across cultures requires further research. Furthermore, because social norms surrounding defecation behavior may also be influenced by the maintenance or cleanliness of a latrine, incorporating additional information on the latrine would add to this exploration and further the generalizability of this research.

## Conclusion

As an often neglected side of the epidemiologic triad, we focus on human behavior at the interface of the environment and biological agent of disease. Our approach, illustrated here for latrine defecation, follows methods commonly used to measure socially stigmatized actions, such as intimate partner violence [64], aggression and bullying [65], or substance abuse [66]. Our work within the WASH sector, which allows us to test common assumptions of how people defecate, a critical component to disease transmission. More research is needed, however, to refine the selection of latrine use indicators. First and foremost, gender specific indicators, which may be different by life course stage [39], will likely provide better insight into population-level drivers of behavior and more accurate classification of latrine users (see our sensitivity analysis in Additional file 4). Second, inconsistent latrine use may have a different set of determinants than consistent latrine use, as these behaviors are not strictly opposites; thus, additional work is required to determine indicators of inconsistent latrine use and whether said indicators can distinguish groups of people. Third, because psychosocial norms, attitudes, and beliefs may change over time, longitudinal analysis are required to determine if these indicators are temporally consistent. Finally, future research is needed to test hypotheses assessing whether latrine use measures are adequate indicators of reduced exposure to enteric pathogens.

Regardless of the disease system of study, epidemiologists interested in population dynamics that lead to health and disease should include metrics of behavior and social process into their research. Development of psychosocial indicators of sensitive behaviors and application of LCA is a useful tool for measurement of sensitive behavioral risk factors, such as hygiene or sexual activity, where self-report data suffer from misclassification and no gold standard measurement exists.

## Additional files

Additional file 1: Polychoric correlations. (DOCX 79 kb)

Additional file 2: Class assignment in each model. (DOCX 78.5 kb)

Additional file 3: Conditional item probabilities for all 16 indicators. (DOCX 28 kb)

Additional file 4: Gender-stratified LCA models. (DOCX 141 kb)

Additional file 5: Imputed latrine use assignment. (DOCX 151 kb)

Additional file 6: R Code for Primary Analysis. (DOCX 92.1 kb)

Additional file 7: Comparison of latrine use metrics and LCA model consistency. (DOCX 150 kb)

## Abbreviations

BIC: Bayesian Information Criterion
CI: Confidence Interval
FIML: Full Information Maximum Likelihood
IBM-WASH: Integrated Behavior Model of Water Sanitation and Hygiene
LCA: Latent Class Analysis
LMICs: Low and Middle-Income Countries
OR: Odds Ratio
SDG: Sustainable Development Goal
SE: Standard Error
WASH: Water, sanitation, and hygiene
