# Supplementary material for: Latent variable modeling to develop a robust proxy for sensitive behaviors: application to latrine use behavior and its association with sanitation access in a middle-income country
Source: BMC Public Health. 2019 Jan 19;19:90. doi: 10.1186/s12889-018-6373-x (PMC6339309; doi:10.1186/s12889-018-6373-x)
Supplement: Supplementary file 1 — Polychoric correlations. (DOCX 79 kb) [file 12889_2018_6373_MOESM1_ESM.docx]

**Additional File 1: Polychoric correlations**

Polychoric correlations, that is correlation between measured, categorical variables, are presented below in Supplementary Table 1. The majority of the indicators have low polychoric correlation. Exceptions of slightly higher correlations (greater than 0.60) include perceived cleanliness of the latrine, strength of the latrine’s basin, and one’s satisfaction with the latrine’s aesthetics. Additionally, convenience of night latrine use has a higher correlation with nighttime safety of the latrine and the strength of the latrine’s basin.

| **Supplementary Table 1**. Polychoric correlations for each indicator included in the study. | | | | | | | | | | | | | | | | |
| --- | --- | --- | --- | --- | --- | --- | --- | --- | --- | --- | --- | --- | --- | --- | --- | --- |
| **Indicators** | When I use the latrine, … | I use the latrine … | I do not use the latrine … | During the dry season, … men…. | During the rainy season, … neighbors … | During the rainy season, ... children … | There are too many people … | If my household did not have its own … | The cabin of … | I am pleased … | The latrine's basin … | The latrine is clean … | It is more convenient to defecate outside … | My morning routine is ... | It is more convenient to use the latrine at night … | It is dangerous … |
| When I use the latrine, … | 1.00 | - | - | - | - | - | - | - | - | - | - | - | - | - | - | - |
| I use the latrine … | 0.30 | 1.00 | - | - | - | - | - | - | - | - | - | - | - | - | - | - |
| I do not use the latrine … | 0.18 | 0.39 | 1.00 | - | - | - | - | - | - | - | - | - | - | - | - | - |
| During the dry season, … men….… | -0.01 | 0.04 | 0.13 | 1.00 | - | - | - | - | - | - | - | - | - | - | - | - |
| During the rainy season, … neighbors … | -0.19 | 0.06 | -0.05 | 0.84 | 1.00 | - | - | - | - | - | - | - | - | - | - | - |
| During the rainy season, ... children … | 0.21 | -0.02 | 0.17 | 0.54 | 0.64 | 1.00 | - | - | - | - | - | - | - | - | - | - |
| There are too many people … | 0.10 | 0.22 | 0.18 | -0.21 | -0.24 | -0.23 | 1.00 | - | - | - | - | - | - | - | - | - |
| If my household did not have its own … | 0.07 | 0.08 | -0.22 | 0.22 | 0.34 | 0.23 | -0.03 | 1.00 | - | - | - | - | - | - | - | - |
| The cabin of … | 0.36 | 0.37 | 0.33 | -0.05 | -0.18 | -0.14 | 0.45 | 0.01 | 1.00 | - | - | - | - | - | - | - |
| I am pleased … | 0.29 | 0.11 | 0.06 | -0.10 | -0.05 | -0.06 | 0.35 | 0.05 | 0.46 | 1.00 | - | - | - | - | - | - |
| The latrine's basin … | 0.06 | 0.31 | 0.24 | -0.07 | 0.06 | -0.10 | 0.35 | 0.22 | 0.28 | 0.83 | 1.00 | - | - | - | - | - |
| The latrine is clean … | 0.33 | 0.27 | 0.14 | -0.01 | 0.14 | 0.25 | 0.11 | 0.30 | 0.39 | 0.62 | 0.66 | 1.00 | - | - | - | - |
| It is more convenient to defecate outside … | 0.04 | 0.31 | 0.06 | -0.07 | -0.10 | 0.12 | 0.07 | 0.11 | 0.19 | 0.17 | 0.32 | 0.43 | 1.00 | - | - | - |
| My morning routine is ... | 0.10 | 0.25 | 0.23 | -0.21 | -0.13 | -0.02 | 0.10 | 0.17 | 0.33 | 0.10 | 0.22 | 0.25 | 0.19 | 1.00 | - | - |
| It is more convenient to use the latrine at night … | 0.07 | 0.24 | 0.38 | 0.16 | 0.02 | -0.04 | 0.11 | -0.01 | 0.27 | 0.48 | 0.62 | 0.34 | 0.39 | 0.15 | 1.00 | - |
| It is dangerous … | 0.25 | 0.07 | 0.33 | 0.11 | 0.14 | 0.19 | 0.17 | 0.03 | 0.17 | 0.35 | 0.53 | 0.44 | 0.20 | 0.22 | 0.68 | 1.00 |
