# Supplementary material for: Latent variable modeling to develop a robust proxy for sensitive behaviors: application to latrine use behavior and its association with sanitation access in a middle-income country
Source: BMC Public Health. 2019 Jan 19;19:90. doi: 10.1186/s12889-018-6373-x (PMC6339309; doi:10.1186/s12889-018-6373-x)
Supplement: Supplementary file 2 — Class assignment in each model. (DOCX 78.5 kb) [file 12889_2018_6373_MOESM2_ESM.docx]

**Additional File 2: Class assignment in each model**

As presented in the main study, the 3-class and 2-class models with all sixteen indicators had comparable predicted class memberships. The class memberships for the 3-class model were “never users” (1%), “sometimes users” (25%), and “always users” (74%) while membership in the 2-class model was “inconsistent latrine users” (22%) or “consistent latrine users” (78%). Using these profile labels, we assume that “never users” and “sometimes users” have the same assignment as “inconsistent users” in the 2-class model. Hence, we aggregated the “never users” and “inconsistent users” into one group so that the model results consisted of two classifications: 26% of the population and 74% of the population. We then compared the assigned classification across the models. Of the 251 people in the study sample, 11 individuals (or 4% of the overall sample) had differing assignments between these models. Those with different assignment were classified as “sometimes users” in the 3-class model, but “consistent users” in the 2-class model. Nevertheless, latrine use assignment was comparable between the models (Cohen’s kappa =0.88).

The 16-indicator 2-class model and the 5-indicator 2-class model had identical overall proportions of predicated class membership (22% vs. 78%). Agreement between models was very high (Cohen’s kappa =0.97), with only three individuals having a different predicted class membership between the two models.
