# Supplementary material for: Latent variable modeling to develop a robust proxy for sensitive behaviors: application to latrine use behavior and its association with sanitation access in a middle-income country
Source: BMC Public Health. 2019 Jan 19;19:90. doi: 10.1186/s12889-018-6373-x (PMC6339309; doi:10.1186/s12889-018-6373-x)
Supplement: Supplementary file 3 — Conditional item probabilities for all 16 indicators. (DOCX 28 kb) [file 12889_2018_6373_MOESM3_ESM.docx]

**Additional File 3: Conditional item probabilities for all 16 indicators**

Final group assignment in an LCA is determined by a set of conditional outcome probabilities of response to each indicator included in the model. Mathematically, this is represented in the following formula, which uses an indicator function so that the probability of 1 is:

$$P\left( Y=y \right)=\sum_{c=1}^{C} \gamma_{c} \prod_{j=1}^{J} \prod_{r_{j}=1}^{R_{j}} \rho_{\left( j, r_{j} | c \right)}^{I\left( y_{j}=r_{j} \right)}$$

In this formula, *j* represents each item and *r_j_* represents the response to the specific item; *c* is the total number of classes. The *γ* parameter is a vector of latent class membership probabilities, which sum to 1. The *ρ* parameter, therefore, is a matrix of item-response probabilities conditional on latent class membership. Overall, the response pattern is *y* (Lanza et al, 2013)^[[1]](#footnote-2)^.

Hence, examination of the conditional item response probability provides insight into how each indicator was used to distinguish between classes. Supplementary Table 3 presents the class-conditional outcome probabilities for each of the 16 items in the 2-class model.

| **Supplemental Table 3.** Parameter estimates for the 2-class model, where the probability of membership in the consistent latrine use class is 0.78 and the probability of membership in the inconsistent latrine use class is 0.22. The item conditional probability and standard error (SE) are presented alongside the corresponding response to the survey question, disaggregated by class membership. | | | | |
| --- | --- | --- | --- | --- |
| **Indicator** | **Consistent Latrine Use** | | **Inconsistent Latrine Use** | |
|  | **Question Response** | **Item Probability (SE)** | **Question Response** | **Item Probability (SE)** |
| When I use the latrine, it causes me to feel anxious.* | No | 0.80 (0.03) | No | 0.84 (0.07) |
| I use the latrine every day.* | Yes | 0.93 (0.02) | Yes | 0.86 (0.08) |
| I do not use the latrine when it is raining because I do not want to get wet.* | Yes | 0.82 (0.03) | Yes | 0.76 (0.09) |
| During the dry season, I think that most of the men in my village regularly use a latrine. | Yes | 0.85 (0.03) | Don't Know | 0.56 (0.14) |
| During the rainy season, I think all of my neighbors regularly use a latrine. | Yes | 0.98 (0.02) | Don't Know | 0.61 (0.14) |
| During the rainy season, I think that most of the children in my village regularly use a latrine. | Yes | 0.95 (0.02) | Yes | 0.61 (0.09) |
| There are too many people in this household for one latrine. | No | 0.52 (0.02) | No | 0.71 (0.07) |
| If my household did not have its own latrine, I would use my neighbor’s latrine. | Yes | 0.90 (0.02) | Yes | 0.72 (0.07) |
| The cabin of the latrine is too small for me to use.* | No | 0.70 (0.04) | No | 0.81 (0.07) |
| I am pleased with how the latrine looks.* | Yes | 0.62 (0.04) | Yes | 0.71 (0.07) |
| The latrine's basin is strong enough to hold my weight.* | Yes | 0.93 (0.02) | Yes | 0.89 (0.04) |
| The latrine is clean enough to use.* | Yes | 0.87 (0.03) | Yes | 0.86 (0.07) |
| It is more convenient to defecate outside than to return home to use the latrine.* | No | 0.54 (0.04) | No | 0.55 (0.10) |
| My morning routine is not suited for using the latrine to defecate.* | No | 0.64 (0.04) | No | 0.72 (0.09) |
| It is more convenient to use the latrine at night than to defecate in a container within my household.* | Yes | 0.87 (0.03) | Yes | 0.77 (0.08) |
| It is dangerous to use the latrine at night.* | No | 0.66 (0.04) | No | 0.57 (0.10) |
| *Uninformative indicator, based on item probability and standard error value for each class | | | | |

1. Lanza, S. T., & Rhoades, B. L. (2013). Latent class analysis: An alternative perspective on subgroup analysis in prevention and treatment. *Prevention Science*, *14*(2), 157-168. [↑](#footnote-ref-2)
