# Supplementary material for: Latent variable modeling to develop a robust proxy for sensitive behaviors: application to latrine use behavior and its association with sanitation access in a middle-income country
Source: BMC Public Health. 2019 Jan 19;19:90. doi: 10.1186/s12889-018-6373-x (PMC6339309; doi:10.1186/s12889-018-6373-x)
Supplement: Supplementary file 4 — Gender-stratified LCA models. (DOCX 141 kb) [file 12889_2018_6373_MOESM4_ESM.docx]

**Additional File 4: Gender-stratified LCA models**

Psychosocial factors influencing latrine use for defecation are likely patterned by gender norms. Thus, any model using these indicators to identify groups of latrine users should be gender-specific. Here, this sensitivity analysis presents gender-stratified LCA models for latrine use for defecation. Overall, the 16 indicators can be used to identify similar groups of consistent and inconsistent latrine users in both strata. Additionally, certain indicators are more useful for predicting latrine use groups among women relative to men and vice-versa. Caution, however, should be used when interpreting these results, as the original study was not sufficiently powered to disaggregate on gender.

In comparing the female-only LCA model (n= 169 women; Supplementary Table 4.1) to the male-only LCA model (n=86 men; Supplementary Table 4.2), a similar proportion of each subpopulation was classified as consistent and inconsistent latrine users. Approximately 80 percent of women and men were grouped as consistent latrine defecation users while about 20 percent of men and women were groups as inconsistent users. Neither of the full LCA models, however, have reasonable model fit per the chi-squared value. This poor fit may be due to unnecessary indicators included in the model, or could also be due to a smaller sample size.

Further examination of the item probability of each indicator and the corresponding standard error suggests that many of the indicators in may be uninformative. Possible informative indicators for the female-only model include:

- During the dry season, I think that most of the men in my village regularly use a latrine.
- During the rainy season, I think all of my neighbors regularly use a latrine.
- During the rainy season, I think that most of the children in my village regularly use a latrine.
- There are too many people in this household for one latrine.
- If my household did not have its own latrine, I would use my neighbor’s latrine.
- The latrine's basin is strong enough to hold my weight.
- It is more convenient to use the latrine at night than to defecate in a container within my household.
- It is dangerous to use the latrine at night.

Of these indicators, the first five were included in the primary analysis as aggregate population proxies of latrine use for defecation. This highlights that women may have unique psychosocial factors influencing their behavior in additional to generic population psychosocial determinants.

In comparison, the indicators in the male-only model that may be driving patterns of latrine use for defecation differ from those observed as important among women as well as in the general population. Note, there are large standard errors for all estimates within the stratified male model, and therefore, the results should be interpreted with caution.

| **Supplementary Table 4.1**. The parameter estimates for the female-only model (n=162) that included all 16 indicators: item conditional probability, standard error (SE), response to the survey question, and whether the indicators was informative for the model. For the female only LCA, the overall probability of assignment into the consistent latrine use membership class is 0.83 while assignment into the inconsistent latrine use class was 0.17. The model fit statistics include BIC = 2,984; X2 = 2.5 E+12; and relative entropy = 0.89. | | | | | |
| --- | --- | --- | --- | --- | --- |
| **Indicator** | **Consistent Latrine Use** | | **Inconsistent Latrine Use** | | **Informative Indicator?** |
|  | **Question Response** | **Item Probability (SE)** | **Question Response** | **Item Probability (SE)** |  |
| When I use the latrine, it causes me to feel anxious. | No | 0.93 (0.08) | No | 0.87 (0.04) | No |
| I use the latrine every day. | Yes | 0.90 (0.14) | Yes | 0.91 (0.03) | No |
| I do not use the latrine when it is raining because I do not want to get wet. | Yes | 0.72 (0.17) | Yes | 0.78 (0.04) | No |
| During the dry season, I think that most of the men in my village regularly use a latrine. | Don't Know | 0.90 (0.03) | Yes | 0.76 (0.05) | Yes |
| During the rainy season, I think all of my neighbors regularly use a latrine. | Don't Know | 0.82 (0.01) | Yes | 0.91 (0.04) | Yes |
| During the rainy season, I think that most of the children in my village regularly use a latrine. | Yes | 0.70 (0.15) | Yes | 0.94 (0.03) | Yes |
| There are too many people in this household for one latrine. | No | 0.76 (0.12) | Yes | 0.56 (0.05) | Yes |
| If my household did not have its own latrine, I would use my neighbor’s latrine. | Yes | 0.72 (0.12) | Yes | 0.90 (0.03) | Yes |
| The cabin of the latrine is too small for me to use. | No | 0.85 (0.08) | No | 0.78 (0.04) | No |
| I am pleased with how the latrine looks. | Yes | 0.68 (0.15) | Yes | 0.64 (0.05) | No |
| The latrine's basin is strong enough to hold my weight. | Yes | 0.84 (0.10) | Yes | 0.95 (0.02) | Yes |
| The latrine is clean enough to use. | Yes | 0.83 (0.10) | Yes | 0.92 (0.03) | Yes |
| It is more convenient to defecate outside than to return home to use the latrine. | No | 0.58 (0.14) | No | 0.61 (0.05) | No |
| My morning routine is not suited for using the latrine to defecate. | No | 0.80 (0.17) | No | 0.74 (0.04) | No |
| It is more convenient to use the latrine at night than to defecate in a container within my household. | Yes | 0.67 (0.18) | Yes | 0.83 (0.05) | Yes |
| It is dangerous to use the latrine at night. | No | 0.54 (0.18) | No | 0.7 (0.06) | Yes |

| **Supplementary Table 4.2**. The parameter estimates for the male-only model (n=89) that included all 16 indicators: item conditional probability, standard error (SE), response to the survey question, and whether the indicators was informative for the model. For the male only LCA, the overall probability of assignment into the consistent latrine use membership class is 0.81 while assignment into the inconsistent latrine use class was 0.19. The model fit statistics include BIC = 1,760; X2 = 8.3 E+12; and relative entropy = 0.91. | | | | | |
| --- | --- | --- | --- | --- | --- |
| **Indicator** | **Consistent Latrine Use** | | **Inconsistent Latrine Use** | | **Informative Indicator?** |
|  | **Question Response** | **Item Probability (SE)** | **Question Response** | **Item Probability (SE)** |  |
| When I use the latrine, it causes me to feel anxious. | No | 0.74 (0.07) | Yes | 0.57 (1.37) | Yes |
| I use the latrine every day. | Yes | 0.92 (0.04) | Yes | 0.94 (13.64) | No |
| I do not use the latrine when it is raining because I do not want to get wet. | Yes | 0.85 (0.05) | Yes | 0.94 (2.65) | No |
| During the dry season, I think that most of the men in my village regularly use a latrine. | Yes | 0.75(0.09) | Yes | 0.84 (49.28) | No |
| During the rainy season, I think all of my neighbors regularly use a latrine. | Yes | 0.82 (0.09) | Yes | 0.88 (52.16) | No |
| During the rainy season, I think that most of the children in my village regularly use a latrine. | Yes | 0.86 (0.06) | Yes | 0.70 (0.90) | No |
| There are too many people in this household for one latrine. | No | 0.69 (0.07) | No | 0.58 (1.73) | No |
| If my household did not have its own latrine, I would use my neighbor’s latrine. | Yes | 0.86 (0.05) | Yes | 0.76 (4.29) | No |
| The cabin of the latrine is too small for me to use. | No | 0.63 (0.07) | No | 0.50 (1.63) | No |
| I am pleased with how the latrine looks. | Yes | 0.77 (0.07) | No | 0.94 (3.48) | Yes |
| The latrine's basin is strong enough to hold my weight. | Yes | 1.00 (0.00) | Yes | 0.58 (1.20) | No |
| The latrine is clean enough to use. | Yes | 0.94 (0.04) | No | 0.70 (0.73) | No |
| It is more convenient to defecate outside than to return home to use the latrine. | Yes | 0.53 (0.07) | Yes | 0.66 (1.44) | No |
| My morning routine is not suited for using the latrine to defecate. | No | 0.51 (0.08) | Yes | 0.51 (1.79) | Yes |
| It is more convenient to use the latrine at night than to defecate in a container within my household. | Yes | 0.98 (0.02) | Yes | 0.66 (1.35) | No |
| It is dangerous to use the latrine at night. | No | 0.69 (0.07) | Yes | 0.82 (0.65) | Yes |
