# Supplementary material for: Latent variable modeling to develop a robust proxy for sensitive behaviors: application to latrine use behavior and its association with sanitation access in a middle-income country
Source: BMC Public Health. 2019 Jan 19;19:90. doi: 10.1186/s12889-018-6373-x (PMC6339309; doi:10.1186/s12889-018-6373-x)
Supplement: Supplementary file 5 — Imputed latrine use assignment. (DOCX 151 kb) [file 12889_2018_6373_MOESM5_ESM.docx]

**Additional File 5: Imputed latrine use assignment**

As described in the main study text, a pseudo-class approach was used to limit any uncertainty that latrine use classification would add to additional statistical analysis. Uncertainty is inherent in the predicted assignments because the true underlying probability distribution of latrine use classes is unknown. In pseudo-class approach, five probabilities of latrine use assignment were imputed. Supplementary Table 5.1 provides the percent distribution of consistent latrine user by sociodemographic characteristics for each of the five imputations.

| **Supplemental Table 5.1**. Population demographics by imputed consistent latrine use assignment. For each imputation, the proportion of the population that are classified as consistent latrine users, by background characteristics, and standard error (SE). | | | | | |
| --- | --- | --- | --- | --- | --- |
| **Demographic Variable** | **Consistent Latrine Use Class (SE)** | | | | |
|  | **Imputation 1** | **Imputation 2** | **Imputation 3** | **Imputation 4** | **Imputation 5** |
| **Age** |  |  |  |  |  |
| Age 13-17 | 0.79 (0.05) | 0.81 (0.05) | 0.79 (0.05) | 0.81 (0.05) | 0.79 (0.05) |
| Age 18-21 | 0.63 (0.07) | 0.64 (0.07) | 0.63 (0.07) | 0.63 (0.07) | 0.69 (0.07) |
| Age 22-26 | 0.82 (0.06) | 0.80 (0.06) | 0.80 (0.06) | 0.80 (0.06) | 0.80 (0.06) |
| Age 27-36 | 0.80 (0.06) | 0.80 (0.06) | 0.80 (0.06) | 0.78 (0.06) | 0.80 (0.06) |
| Age 37-83 | 0.80 (0.06) | 0.82 (0.06) | 0.80 (0.06) | 0.82 (0.06) | 0.80 (0.06) |
| **Sex** |  |  |  |  |  |
| Females | 0.75 (0.04) | 0.75 (0.04) | 0.74 (0.04) | 0.75 (0.04) | 0.75 (0.04) |
| Males | 0.80 (0.04) | 0.82 (0.06) | 0.81 (0.04) | 0.81 (0.04) | 0.82 (0.06) |
| **Ethnicity** |  |  |  |  |  |
| Afro-Ecuadorian | 0.76 (0.03) | 0.77 (0.03) | 0.75 (0.03) | 0.76 (0.03) | 0.76 (0.03) |
| Mestizo and Other | 0.79 (0.07) | 0.79 (0.07) | 0.79 (0.07) | 0.76 (0.07) | 0.79 (0.07) |
| Chachi | 0.77 (0.05) | 0.79 (0.05) | 0.79 (0.05) | 0.80 (0.05) | 0.82 (0.05) |
| **Educational Attainment** |  |  |  |  |  |
| Less than primary school | 0.70 (0.06) | 0.71 (0.06) | 0.70 (0.06) | 0.71 (0.06) | 0.70 (0.06) |
| Completed primary school | 0.76 (0.06) | 0.75 (0.06) | 0.75 (0.06) | 0.75 (0.06) | 0.76 (0.06) |
| Less than secondary school | 0.78 (0.04) | 0.80 (0.04) | 0.79 (0.04) | 0.80 (0.04) | 0.82 (0.04) |
| Completed secondary school | 0.84 (0.06) | 0.84 (0.06) | 0.81 (0.06) | 0.79 (0.06) | 0.79 (0.06) |
| **Payment Type Received for Employment** |  |  |  |  |  |
| Solely cash | 0.81 (0.04) | 0.81 (0.04) | 0.81 (0.04) | 0.81 (0.04) | 0.80 (0.04) |
| Cash and kind/ solely kind | 0.88 (0.08) | 0.88 (0.08) | 0.81 (0.10) | 0.88 (0.08) | 0.94 (0.06) |
| Not paid | 0.73 (0.07) | 0.76 (0.07) | 0.78 (0.06) | 0.76 (0.07) | 0.78 (0.06) |
| Not employed in the previous 12 months | 0.71 (0.05) | 0.71 (0.05) | 0.68 (0.05) | 0.70 (0.05) | 0.71 (0.05) |
| **Living in Household with Cement Walls** |  |  |  |  |  |
| Yes | 0.74 (0.04) | 0.74 (0.04) | 0.73 (0.04) | 0.74 (0.04) | 0.74 (0.04) |
| No | 0.82 (0.04) | 0.83 (0.04) | 0.82 (0.04) | 0.73 (0.04) | 0.83 (0.04) |
| **Living in an Asset-Deprived Household** |  |  |  |  |  |
| Yes | 0.72 (0.04) | 0.75 (0.04) | 0.74 (0.04) | 0.74 (0.04) | 0.73 (0.04) |
| No | 0.80 (0.04) | 0.80 (0.04) | 0.77 (0.04) | 0.79 (0.04) | 0.74 (0.04) |

The regression model results for each imputed outcome are presented in Supplementary Tables 5.2 (unadjusted) and 5.3 (adjusted). Of note, overall results reflect combination of each of these model following Rubin’s rules.

| **Supplementary Table 5.2**. Unadjusted association between access to basic sanitation and latrine use. Each of the five models uses imputed latrine use as the outcome. The odds ratio (OR) for basic sanitation access, along with the 95% confidence intervals (CI), is presented. The combined odds ratio between household access to basic sanitation and consistent latrine use is 1.1 (95% CI = 0.56-2.2, *within-model variance = 0.092, **between model variance = 0.024) | | | |
| --- | --- | --- | --- |
| **Individual models** | **OR** | **Lower Boundary** | **Upper Boundary** |
| Model 1: Basic Sanitation | 1.1 | 0.6 | 1.9 |
| Model 2: Basic Sanitation | 1.1 | 0.6 | 1.9 |
| Model 3: Basic Sanitation | 1.2 | 0.7 | 2.2 |
| Model 4: Basic Sanitation | 1.2 | 0.6 | 2.1 |
| Model 5: Basic Sanitation | 1.1 | 0.6 | 1.9 |
| *an average of the variance across imputations | | | |
| **[1/(m-1]*(imputed model coefficient-average coefficient)^2; m= number of imputations | | | |

| **Supplementary Table 5.3**. Adjusted association between access to basic sanitation and latrine use. Each of the five models uses imputed latrine use as the outcome. The odds ratio (OR) for each variable included in the model, along with the 95% confidence intervals (CI), is presented. The summary within-model and between-model variance is also presented. | | | | | | | |
| --- | --- | --- | --- | --- | --- | --- | --- |
|  | **Adjusted Models with Imputed Latrine Use Outcomes** | | | | | ***Within-Model Variance** | ****Between-Model Variance** |
| **Model Covariate** | **1. OR (95% CI)** | **2. OR (95% CI)** | **3. OR (95% CI)** | **4. OR (95% CI)** | **5. OR (95% CI)** |  |  |
| Less than basic sanitation *(referent group)* | 1 (-) | 1 (-) | 1 (-) | 1 (-) | 1 (-) | - | - |
| Basic sanitation | 1.0 (0.55-2.0) | 0.95 (0.51-1.8) | 1.1 (0.60-2.2) | 1.2 (0.62-2.3) | 1.0 (0.55-1.9) | 0.11 | 0.01 |
| Less than primary school *(referent group)* | 1 (-) | 1 (-) | 1 (-) | 1 (-) | 1 (-) | - | - |
| Completed primary school | 1.6 (0.68-3.6) | 1.3 (0.54-3.0) | 1.3 (0.59-3.0) | 1.3 (0.57-2.9) | 1.5 (0.64-3.7) | 0.18 | 0.01 |
| Less than secondary school | 1.9 (0.86-4.0) | 2.1 (0.94-4.8) | 1.9 (0.90-4.2) | 2.1 (0.93-4.8) | 2.5 (1.1-5.8) | 0.17 | 0.01 |
| Completed secondary school | 3.1 (1.1-8.9) | 2.6 (0.98-7.0) | 2.3 (0.92-5.9) | 1.9 (0.74-4.8) | 2.1 (0.84-5.5) | 0.24 | 0.04 |
| Chachi, Mestizo, or Other Ethnicity (*referent group*) | 1 (-) | 1 (-) | 1 (-) | 1 (-) | 1 (-) | - | - |
| Afro-Ecuadorian | 1.2 (0.65-2.2) | 1.0 (0.54-1.9) | 0.94 (0.50-1.8) | 1.0 (0.56-1.9) | 0.91 (0.48-1.7) | 0.10 | 0.01 |
| Male *(referent group)* | 1 (-) | 1 (-) | 1 (-) | 1 (-) | 1 (-) | - | - |
| Female | 1.3 (0.68-2.5) | 1.7 (0.82-3.4) | 1.5 (0.79-3.0) | 1.6 (0.84-3.2) | 1.6 (0.79-3.3) | 0.12 | 0.01 |
| Household walls constructed of other material (*referent group*) | 1 (-) | 1 (-) | 1 (-) | 1 (-) | 1 (-) | - | - |
| Household constructed of cement | 0.49 (0.24-1.0) | 0.63 (0.32-1.2) | 0.68 (0.35-1.3) | 0.63 (0.32-1.2) | 0.56 (0.28-1.1) | 0.12 | 0.02 |
| Asset Deprived Household (*referent group*) | 1 (-) | 1 (-) | 1 (-) | 1 (-) | 1 (-) | - | - |
| Non-asset Deprived Household | 0.48 (0.24-1.0) | 0.47 (0.24-0.91) | 0.50 (0.26-1.0) | 0.54 (0.27-1.1) | 0.46 0.24-0.89) | 0.12 | 0.004 |
| *an average of the variance across imputations | | | | | | | |
| **[1/(m-1]*(imputed model coefficient-average coefficient)^2; m= number of imputations | | | | | | | |
|  | | | | | | | |
