# Supplementary material for: Latent variable modeling to develop a robust proxy for sensitive behaviors: application to latrine use behavior and its association with sanitation access in a middle-income country
Source: BMC Public Health. 2019 Jan 19;19:90. doi: 10.1186/s12889-018-6373-x (PMC6339309; doi:10.1186/s12889-018-6373-x)
Supplement: Supplementary file 6 — R Code for Primary Analysis. (DOCX 92.1 kb) [file 12889_2018_6373_MOESM6_ESM.docx]

**Additional File 6: R Code for primary analysis**

library(poLCA)

library(survey)

library(polycor)

####---Polychoric Correlations---####

#LCA dataset

LCA <-subset(INDIVID, select=c(ID,P37,P38,P39,P40,P41,P42,P43,P44,P45,P47,P48,P49,P50,P51,P52,P53))

LCA <-LCA[order(LCA$ID),]

LCA[LCA==0]<-2

LCA[is.na(LCA)]<-3

summary(LCA)

#Correlations

Cor <-subset(LCA, select = -c(ID))

Cor[sapply(Cor, is.numeric)] <-lapply(Cor[sapply(Cor, is.numeric)],

as.factor)

str(Cor)

hetcor(Cor, ML = FALSE, std.err = TRUE, pd=TRUE)

####---LCA Models---####

##Function for LCA

f <-with(LCA, cbind(P37,P38,P39,P40,P41,P42,P43,P44,P45,P47,P48,P49,P50,P51,P52,P53)~1)

##Function for Relative Entroy

entropy <-function (p) sum(-p*log(p))

##LCA Models for Comparison

lc2 <-poLCA(f, data=LCA, nclass=2, nrep=30, na.rm=FALSE, maxiter=50000, graph=TRUE)

lc2$posterior <-(lc2$posterior+0.00001)

error_prior <-entropy(lc2$P) # Class proportions

error_post <-mean(apply(lc2$posterior, 1, entropy))

R_entropy_2 <-(error_prior - error_post) / error_prior

lc3 <-poLCA(f, data=LCA, nclass=3, nrep=30, na.rm=FALSE, maxiter=50000, graph=TRUE)

lc3$posterior <-(lc3$posterior+0.00001)

error_prior <-entropy(lc3$P) # Class proportions

error_post <-mean(apply(lc3$posterior, 1, entropy))

R_entropy_3 <-(error_prior - error_post) / error_prior

###LCA with Limited Dataset (based on item response)

f.limited <-with(LCA, cbind(P40,P41,P42,P43,P44)~1)

lc2.limited <-poLCA(f.limited, data=LCA, nclass=2, nrep=30, na.rm=FALSE, maxiter=50000, graph=TRUE)

lc2.limited$posterior <-(lc2.limited$posterior+0.00001)

error_prior <-entropy(lc2.limited$P) # Class proportions

error_post <-mean(apply(lc2.limited$posterior, 1, entropy))

R_entropy_2_limited <-(error_prior - error_post) / error_prior

####---Multiple Imputation Approach for Predicted Class Membership---####

#Class 1: Consistent Users

#Class 2: Inconsistent User

posterior <-data.frame(lc2.limited$posterior)

lcaprobs <-cbind(posterior, lc2.limited$predclass, LCA$ID)

names(lcaprobs)[names(lcaprobs) == 'lc2.limited$predclass'] <- 'class.assign'

names(lcaprobs)[names(lcaprobs) == 'LCA$ID'] <- 'ID'

lcaprobs$class.predict<-ifelse(lcaprobs$X1>=0.5, 1, 2)

tab1(lcaprobs$class.predict)

table(lcaprobs$class.predict, lcaprobs$class.assign) #Note: class assignment is estimates from probability of 1 <=50%

#Random Draw from Uniform Distribution

lcaprobs$u1 <- runif(251)

lcaprobs$u2 <- runif(251)

lcaprobs$u3 <- runif(251)

lcaprobs$u4 <- runif(251)

lcaprobs$u5 <- runif(251)

#Assign Class Membership based on random draw value

lcaprobs$class1[lcaprobs$u1 <= lcaprobs$X1] <- 1

lcaprobs$class1[lcaprobs$u1 > lcaprobs$X1] <- 2

tab1(lcaprobs$class1)

lcaprobs$class2[lcaprobs$u2 <= lcaprobs$X1] <- 1

lcaprobs$class2[lcaprobs$u2 > lcaprobs$X1] <- 2

tab1(lcaprobs$class2)

lcaprobs$class3[lcaprobs$u3 <= lcaprobs$X1] <- 1

lcaprobs$class3[lcaprobs$u3 > lcaprobs$X1] <- 2

tab1(lcaprobs$class3)

lcaprobs$class4[lcaprobs$u4 <= lcaprobs$X1] <- 1

lcaprobs$class4[lcaprobs$u4 > lcaprobs$X1] <- 2

tab1(lcaprobs$class4)

lcaprobs$class5[lcaprobs$u5 <= lcaprobs$X1] <- 1

lcaprobs$class5[lcaprobs$u5 > lcaprobs$X1] <- 2

tab1(lcaprobs$class5)

####---Regression Model---####

load("LCA_cross.Rdata")

summary(LCA_cross)

##Design: Accounting for HH-level Clustering

design <-svydesign(id=~HH_ID, data=LCA_cross)

##JMP_Class: Unadjusted

summary(svyglm(class1~as.factor(JMP_class), family=binomial, design=design))

summary(svyglm(class2~as.factor(JMP_class), family=binomial, design=design))

summary(svyglm(class3~as.factor(JMP_class), family=binomial, design=design))

summary(svyglm(class4~as.factor(JMP_class), family=binomial, design=design))

summary(svyglm(class5~as.factor(JMP_class), family=binomial, design=design))

##JMP_Class: Categorical education + Race + Gender + Wall + Land

summary(svyglm(class1~as.factor(JMP_class)+as.factor(edu_cat)+race+Gender+asset+wall, family=binomial, design=design))

summary(svyglm(class2~as.factor(JMP_class)+as.factor(edu_cat)+race+Gender+asset+wall, family=binomial, design=design))

summary(svyglm(class3~as.factor(JMP_class)+as.factor(edu_cat)+race+Gender+asset+wall, family=binomial, design=design))

summary(svyglm(class4~as.factor(JMP_class)+as.factor(edu_cat)+race+Gender+asset+wall, family=binomial, design=design))

summary(svyglm(class5~as.factor(JMP_class)+as.factor(edu_cat)+race+Gender+asset+wall, family=binomial, design=design))
