# Supplementary material for: Latent variable modeling to develop a robust proxy for sensitive behaviors: application to latrine use behavior and its association with sanitation access in a middle-income country
Source: BMC Public Health. 2019 Jan 19;19:90. doi: 10.1186/s12889-018-6373-x (PMC6339309; doi:10.1186/s12889-018-6373-x)
Supplement: Supplementary file 7 — Comparison of latrine use metrics and LCA model consistency. (DOCX 150 kb) [file 12889_2018_6373_MOESM7_ESM.docx]

**Additional File 7: Comparison of latrine use metrics and LCA model consistency**

While it is not possible to validate the latent variable classification, we can gain insight into the difficulty of measuring latrine use by comparing three different measurement approaches: frequency of latrine use, calculated latrine use (from self-report of all defecation practices within the last week), and latent variable classification. Frequency of latrine use was measured by asking one survey question, with three possible response options (always, sometimes, or never): How frequently do you use a latrine? Calculated latrine use was measured through responses to a series of 20 survey questions. First, each respondent was asked about 10 common defecation behaviors and whether anyone in the community practiced each defecation behavior (response was binary, yes/no). Then, the respondent was asked how many times in the last week they individually practiced each of these 10 defecation practices, with probing included for defecation in the morning, afternoon, evening, and night for each specific behavior. If a respondent reported at least one defecation behavior that was not latrine use in the past week, we classified them as “inconsistent” latrine use. Otherwise, they were considered an “always” latrine user. Supplementary Table 7.1 presented the percent of the total population that is a latrine user according to each of these different calculations.

| **Supplementary Table 7.1.** Percentage of the Population Reporting Latrine Use via Two Different Survey Approaches | | | |
| --- | --- | --- | --- |
| **Latrine Use Variable** | **Total Sample (n=251)** | **Women (n=162)** | **Men (n=89)** |
| **Frequency of Latrine Use** |  |  |  |
| Always | 0.89 | 0.91 | 0.87 |
| Frequently | 0.10 | 0.08 | 0.12 |
| Never | 0.01 | 0.01 | 0.01 |
| **Calculated Latrine Use** |  |  |  |
| Consistent | 0.72 | 0.82 | 0.53 |
| Inconsistent | 0.28 | 0.18 | 0.47 |

As evident in Supplementary Table 7.1, even though both variables were estimated by self-report responses to questions about personal defecation practices, there are notable differences between those that reported always using a latrine. A greater proportion of the sample self-reported always using a latrine use in the frequency-style survey question relative to the caluated approach. While these differences were evident in the total population, greater difference appear among men, where approximately half of men interviewed reported only use a latrine for defecation in the previous week (and nearly 9 in 10 reported always using a latrine). This pattern is not suprising, for we also collected qualitative data alongside the quantitative interviews, which showed several cases of discrepant answers throughout the various interviews. For example, some individuals that were interviewed with the quantitative survey reported always using a latrine also discussed discussed various forms of open defecation they practiced (such as defecation in the river or in a leaf) during the qualitative interviews. Additionally, husbands and wives living in the same household (who were interviewed separately) discussed each other’s behaviors during the qualitative interviews, which differed from the partner’s self-reported defecation behaviors. The overall distinction in response patterns to the self-reported questions, together with to the qulatitive interview responses, provide additional evidence of likely over-reporting of latrine use behavior.

In addition to observing the overall distribution of latrine use via each different measuremen approach, we can also assess the agreement between each of the three variables - frequency of latrine use, calculated latrine use, and latent variable classification (Supplementary Table 7.2). By using Fleiss’ kappa statistic to assess agreement, the three variables exhibit slight agreement in the total sample as well as in women and men. No more than slight agreement was observed between all combinations of the variables, with the kappa statistic ranging from 0.07 to 0.15. Overall, the women included in the sample showed marginally better agreement between the variables relative to men and women. The slight agreement between the three variables provides further rationale that validation with self-reported latrine use should be not conducted.

| **Supplementary Table 7.2.** Agreement between each approach to measuring latrine use | | | |
| --- | --- | --- | --- |
| **Agreement between the following variables (kappa statistics)** | **Total Sample** | **Women** | **Men** |
| Frequency of latrine use, calculated latrine use, and latent variable classification* | 0.08 | 0.1 | 0.03 |
| Frequency of latrine use and calculated latrine use | 0.15 | 0.17 | 0.11 |
| Frequency of latrine use and latent variable classification | 0.06 | 0.17 | 0.16 |
| Calculated latrine use and latent variable classification | 0.07 | 0.15 | 0.02 |
| *Fleiss' kappa where all other comparisons are calculated with Cohen's kappa | | | |

The agreement comparison suggests that each of that while these variables may be measuring the same constructs, they are each subject to different types of error. The error generated from the latent variable classification of latrine use is reflected in the model entropy value (0.86), while the error in self-reported latrine use cannot be quantified. If the latent classification was free of measurement error, the entropy value would be 1.0, which is unrealistic in any survey-based research. Nonetheless, we are interested in providing additional evidence that the latent classifications are reliable and overall not driven by certain observations within the dataset. To assess the extent to which the latent classifications are robust, we compared the model results from the full sample to the same model run in 10 randomly selected subsamples of the dataset (Supplementary Table 7.3). Overall, the models run in the randomly selected subsamples showed little variability (for example, entropy ranged from 0.84 to 0.92). The average fit statistics from the subsample models were very comparable to the model with the full dataset.

| **Supplementary Table 7.3.** LCA models in 10 subsamples of the overall dataset (n=225 in each sample). The average fit statistics for each of these 10 models are: BIC =1365; X2=381; entropy=0.87. Overall, the 10 subsamples have an average predicted class membership of 0.78 and 0.22. | | | | |
| --- | --- | --- | --- | --- |
| **Subsample** | **BIC** | **X^2^** | **Relative entropy** | **Predicted class probabilities** |
| Sample 1 | 1392 | 395 | 0.86 | 0.75; 0.25 |
| Sample 2 | 1354 | 363 | 0.86 | 0.78; 0.22 |
| Sample 3 | 1368 | 465 | 0.92 | 0.78; 0.22 |
| Sample 4 | 1361 | 240 | 0.85 | 0.78; 0.22 |
| Sample 5 | 1363 | 370 | 0.88 | 0.78; 0.22 |
| Sample 6 | 1338 | 353 | 0.85 | 0.77; 0.23 |
| Sample 7 | 1332 | 457 | 0.88 | 0.80; 0.20 |
| Sample 8 | 1378 | 372 | 0.84 | 0.78; 0.22 |
| Sample 9 | 1378 | 411 | 0.87 | 0.77; 0.23 |
| Sample 10 | 1389 | 382 | 0.88 | 0.77; 0.23 |

In aggregate, these sensitivity analyses (Supplementary Tables 6.1-6.3), add to our conclusions that the LCA model adequately distinguished the underlying probability of latrine use through use of psychosocial variables. With the ability to assess error in the final latent variable classifications, we also suggest that our models are minimally biased relative to the bias that would be included in self-reported latrine use variables.
